# Creating a Composite Index to Target Recruitment of UK Students from Areas of Low Participation in Higher Education

**DOI:** 10.1007/s12061-021-09423-1

**Published:** 2021-08-17

**Authors:** Douglas Bell, Luke Burns

**Affiliations:** School of Geography, University of Leeds, Leeds, LS2 9JT UK

**Keywords:** Widening participation, Higher education, Composite indicators, Deprivation indices, Spatial analysis, Deprivation indices

## Abstract

This research presents a framework through which a new Higher Education Access and Deprivation (HEAD) composite index was created to assist UK universities in efficiently recruiting and supporting students from areas with traditionally low engagement rates in higher education. The index was designed to be easily adaptable by university staff to suit their own work priorities and/or an institution’s strategic requirements by utilising open socio-demographic data and construction techniques that require minimal technical statistical skills. Although Cornwall was used as the study area in this research, this index has been designed such that it can be readily applied elsewhere. Two differently weighted models were created using the framework – one with equal weights and the other based on the frequency the constituent indicators appeared in the reviewed academic literature. Both models were mapped across Cornwall, identifying areas of deprivation at a finer resolution than under the current widely used Participation of Local Areas (POLAR) methodology. The weighted model performed marginally better than both the equal weighted model and the current POLAR methodology when validated against external data, and additionally it worked well in both rural and urban environments leading to it being selected as the new HEAD index. The HEAD index maps smaller scale areas of deprivation than previously available, and by enabling users to investigate the underlying socio-demographic characteristics of an area, it also allows universities to create interventions, support, and policies that best targets the students they aim to recruit and teach.

## Introduction

Increasing the proportion of university students from disadvantaged areas is a core pillar of UK higher education (HE) policy as seen in the current government’s strategy emphasis on Access and Participation (DfE, 2018). This evolved from the Widening Participation (WP) policies of the previous Labour administrations which set an ambitious target of 50% of young people progressing from school to university, with specific emphasis placed on attracting students from previously under-represented groups such as lower social classes, ethnic minorities, and students with disabilities (House of Commons Education and Skills Committee, 2003). A higher level of education is seen as having both personal and societal benefits, with graduates estimated to earn on average 20% more over their lifetimes than peers who did attend university, while also generating increased tax revenue for the country (Britton et al., 2020). Additionally, having an educated and skilled workforce is regarded by the government as key to increasing social mobility, boosting the country’s productivity and vital for increasing growth in the science, education and digital economic sectors (DfE, 2017).

Since the early days of WP, universities have been encouraged to recruit students from areas with low higher education progression through staff-run outreach projects and interventions such as residential summer schools, in-school aspiration-raising talks, visits by pupils onto campus for taster days and the production of promotional literature. Currently national and regional coordination of outreach activity is organised by the Office for Students under the Uni Connect banner (OfS, 2019b).

The targeting of students from these areas has largely been undertaken with the creation of the Participation of Local Areas (POLAR) spatial dataset, which identifies “low-participation neighbourhoods” or LPNs. POLAR is based on the Young Participation Rate (YPR) measure which shows the percentage of school pupils from a given area who progress to university. For each area, a total annual secondary school cohort size is calculated by using census and Child Benefit data. The number of students progressing from that cohort to HE is then determined by matching records across several data sources, including student application records from the University and Colleges Admissions Service (UCAS) and university enrolment records from the Higher Education Statistics Agency (HESA). By following this process it is then possible to calculate, for a given neighbourhood, the percentage of students progressing onward from school to university, i.e. the YPR (HEFCE, 2005b). YPR scores for each area are then ranked from the lowest to highest and split into quintiles, with the quintile number becoming that area’s POLAR value; a value of 1 means the area belongs to the 20% of locations with the lowest HE participation rates in the UK, while those with a value of 5 are in the 20% with the highest participation rates. Low Participation Neighbourhoods are defined as areas with POLAR scores of 1 or 2. Currently, Uni Connect has identified and prioritised 997 target areas for intervention using POLAR data (OfS, 2020b).

Since its general release in 2005, POLAR’s use has become endemic in the UK HE sector largely through being linked to funding mechanisms. Between 2008/09 and 2010/11 a total of £353.4 million of additional funding was allocated to universities based on their recruitment of students from LPNs (HEFCE, 2008b, 2009, 2010b). POLAR remains important today as universities are required to submit Access and Participation Plans that prove their commitment to diversifying their student body to the Office for Students (OfS) as a condition for charging their students the full 2020/21 annual fee rate of £9,250 (OFFA, 2018). The OfS “strongly encourages providers” to use POLAR within these plans to benchmark and track their progress (OfS, 2019a).

Whilst the 50% target of young people progressing to university was finally achieved in 2018 (DfE 2019), closer examination of the data reveals that access and participation inequalities persist across many demographic groups, e.g. male HE participation was 44.1% in 2017/18 compared to 56.6% for the female population. To this end, the Access and Participation agenda still identifies specific target groups including ethnic minorities, students with disabilities, and those from disadvantaged backgrounds including care leavers and working class young white males (DfE, 2018). The identification of LPNs through POLAR does not identify locales where these demographic groups exit, but where HE progression is low. In contrast, this research presents a framework from which a new Higher Education Access and Deprivation index (HEAD) was developed using open-source demographic data to identify areas of under-representation based on factors that are likely to impact educational progression in the UK. The measure seeks to simplify an inherently multidimensional and complex concept through combining a range data with scientifically proven links to Higher Education access. Using techniques requiring minimal technical skills, the developed framework can be easily adapted by university administrators and outreach staff to their required recruitment catchment areas and/or to meet their institution’s own strategic requirements in recruiting and supporting students from disadvantaged areas. Whilst the work presented in this paper is a first-attempt at such an index, it also acts as a prototype with a revised index incorporating newer data, such as from the 2021 national census, to follow when such data are available.

### Reviewing Spatial Analysis and Higher Education Access

POLAR’s targeted approach places widening participation firmly alongside other Area-Based Initiatives (ABIs) popularised by the Labour government in the late 1990s/early 2000s to efficiently distribute public funds to where they are most needed. Lively debate from that period questioned their efficiency over individual or national interventions as the “measurable characteristics of the neighbourhood add little to our ability to explain variation in outcomes” (McCulloch, 2001, p.681). Responses were varied and included arguments that neighbourhoods are not defined by arbitrary census geography, that ABIs were both a political and pragmatic solution to difficult social problems and that they can exist alongside national/individual interventions (Dorling et al., 2001). The tensions raised in this debate remain as seen in the responses to the use of POLAR to target ‘non-traditional’ student recruitment.

Geography is placed firmly at the centre of the POLAR methodology as “advantaged and disadvantaged groups are defined by where young people live” (HEFCE, 2005b, p.9) although some voices argue that without a basic understanding of its production and the inbuilt assumptions that the measure carries may have led to its over-use or misuse within the sector (Harrison & McCaig, 2015; Holland et al., 2017). Perhaps the biggest assumption is that small areas are homogenous, which can lead to users of measures such as POLAR jumping to the wrong conclusions about individuals from those areas based on aggregated data, i.e. the ecological fallacy. By not recognising this and by over-relying on POLAR as a sole diagnostic tool may lead to disadvantaged students living in affluent areas missing out on assistance, while less deprived students living in poorer areas may gain advantage (Harrison & McCaig, 2015). This simplistic and under-theorised approach to geography may do little to address underlying social problems (Rees et al., 2007).

Concerns over homogeneity can partially be resolved by switching to a finer resolution spatial unit. Census Area Statistic (CAS) Wards were used in the first three POLAR data releases, with the Middle Layer Super Output Area (MSOA) becoming the preferred geographical unit in POLAR4 (Table 1). This choice of scale was set to provide “a balance between resolution in identifying advantaged and disadvantaged areas and the reliability of the ranking statistic” (HEFCE, 2005b, p.52). However, users have reported this as being too broad for targeted outreach work, with further research showing large proportions of lower socio-economic households existing in areas not classified as LPNs under POLAR (Harrison & Hatt, 2010, p.84). The report accompanying the launch of POLAR3 included results of research conducted at sub-ward level across ten cohorts as a response to such criticisms, and conceded that while pockets of deprivation did exist within areas of greater higher education participation it disputed that the level was unacceptable as it affected only 1 in 14 disadvantaged young people (HEFCE, 2014).

**Table 1:** Key features of POLAR data releases (HEFCE, 2005a, 2008a, 2014, 2017)

Release	Date	Geography used	Cohort Used
POLAR1	2002 to closed group. 2005 general release to public	1998 Enumeration Districts or 1991 Census Wards	Children ending secondary education between 1994/95 and 1996/97 and entered HE aged 18 or 19 between 1997 and 1999
POLAR2	2008	Census Area Statistics ward (CAS wards)	Updated to HE Entrants aged 18 or 19, between academic years 2000/01–2005/06
POLAR3	2014	Census Area Statistics ward (CAS wards)	Updated to HE entrants aged 18 between 2005/06–2009/10 or aged 19 between 2006/07–2010/11
POLAR4	2017	Middle Super Output Area (MSOA)	15 yr olds who started secondary school between 2006–2011 and entered HE between 2009/10–2013-14 aged 18, or between 2010/11–2014/15 aged 19

Closely related to the questions around homogeneity is the modifiable areal unit problem (MAUP), as boundaries such as the MSOA are artificially created to support data collection and/or administrative tasks rather than reflecting real life (Harrison & Hatt, 2010; Harrison & McCaig, 2015; Singleton, 2010). As noted by Burrows and Bradshaw (2001, p.1347) “Wards are not neighbourhoods”, and as a consequence no matter what dataset, scale, or boundaries are used for targeting “all are flawed in some way” (Allen, 2010, p.140).

Other concerns raised include that geographical indicators provide practitioners with little assistance in identifying academic potential when targeting individual students (Tate et al., 2011), with local knowledge often being overlooked by the use of POLAR (Harrison & McCaig, 2015). Further the collection, sharing and safeguarding of the data necessary to effectively target under-represented students is problematic due to its sensitive nature and the fragmented nature of the educational system (Holland et al., 2017), especially when there is the additional potential to stigmatize students from LPNs (Harrison & McCaig, 2015). Additional moral and ethical concerns include the omission of voices from disadvantaged communities from the original development of WP policy (Greenbank, 2006), and the use of recruiting disadvantaged students solely to achieve recruitment targets rather as a means to achieve genuine access and participation for all (Coates & Adnett, 2003).

While much of the response to POLAR appears negative, it is accepted that “‘good’ targeting is an essential prerequisite for widening participation” (Harrison & Hatt, 2010, p.66) and that educational ABIs “have been successful at making things better than they would otherwise be” (Rees et al., 2007, p.271).

### Data reduction Techniques and Higher Education

Geodemographic classifications and composite indices are two alternative methodologies that accommodate the input of multiple datasets in spatial analysis. These can be used to overcome the limitation of POLAR by not considering other factors that can cause disengagement from higher education progression.

Geodemographics is defined as an “attempt to characterise people by where they live” (Batey et al., 1999, p.282). Historically, geodemographic classifiers have leaned heavily on traditional census data, however as censuses prove increasingly costly, administrative, survey and/or big data have supplemented their production and are likely to usurp census data as the primary data source (Leventhal, 2016). Whatever their origin, a range of subject-appropriate geocoded variables are selected, with data then sourced, cleansed, and standardised prior to processing. A clustering algorithm is then used to identify groupings or areas with underlying similarities which are then named based on their characteristics, e.g. CACI’s A Classification of Residential Neighbourhoods (ACORN) currently includes area classification clusters such as “Deprived areas and high-rise flats”, “Post-war estates limited means” and “Exclusive Enclaves” (2014). Pen portraits detailing what a typical resident or households looks like for each named group are then often created to provide an understandable qualitative description for non-expert users.

The use of geodemographics in recruitment of university students has been examined by Tonks and Farr (1995) and Batey et al. (1999). All overtly concluded their methodology was consistent with the government’s ABI approach. Singleton (2010) analysed participation rates by geodemographic group showing it was possible to segment data down to the individual subject level for outreach and marketing purposes. Similar work also designed a geodemographic classification for the higher education sector (Singleton & Longley, 2009), and has repeatedly explored the use of geodemographics within higher education in a variety of contexts (Singleton, 2009, 2012; Singleton et al., 2012; Thiele et al., 2016).

The same datasets used in geodemographic classifications are equally suited in composite index construction, as the two methodologies only diverge after the data is cleansed and standardised. Instead of clustering, describing, and naming the groupings, with composite indicators the contributing variables or domains are aggregated into one numerical measure, sometimes with the assistance of weightings to prioritise variable importance. The resulting index can then be used to judge the relative position of one area against its neighbours and to track their changes in position over time (OECD, 2008).

Spatial composite indices remain under-utilised in higher education for outreach, although they have been used to rank research or overall university performance (Asif & Searcy, 2014; El-Hefnawy et al., 2014; Hicks, 2009). The nearest comparable measure discovered was UCAS’ Multiple Equality Measure (MEM) created to assess the diversity of undergraduate applicants to UK universities (UCAS, 2018), built from six variables including gender, POLAR3 quintile, ethnic group, free school meals, school type and Indices of Multiple Deprivation Ranking (IMD), using a mix of UCAS application data and National Pupil Database records. To date, the use of MEM’s has been limited to providing an overview for end-of-cycle reporting on equality (UCAS, 2019) rather than as a spatial analysis tool, although with the inclusion of POLAR3 quintile and IMD ranking, and with postcode being a required field on university applications, it would require little additional effort to operationalise this spatially. There is no reason why composite indices cannot be used to analyse participation in HE as the methodology has been used to classify other forms of deprivation both locally (Burke & Jones, 2019) and nationally (ONS, 2019a), as well as having proven adaptable to a wide array of study topics including assessing government quality (Charron et al., 2015), the integration of land use with transport (Dur & Yigitcanlar, 2015), exploring the risk to agriculture from climate change (Wiréhn et al., 2015) or modelling loneliness in elderly people (Lucy & Burns, 2017).

As both composite indices and geodemographics rely on spatial data, they suffer from the same intrinsic problems as POLAR. Such problems include scale, spatial heterogeneity, the ecological fallacy, and modifiable areal unit problem, with each of these limitations discussed previously and further explored in related works, e.g. Saib et al. (2015), Siegel et al. (2016) and Singleton (2010). Furthermore, any classification does not remove the potential for misuse or stigmatism of students which could be heightened through the use pen portraits which assign names to clusters of areas in geodemographic classifications. Walheer (2018, p.895) states that composite indices “are easy to construct and to interpret” without the need to understand the operation and assumptions built into complex geodemographic clustering algorithms and their results. Dur and Yigitcanlar (2015) further highlight their strength in reducing complex information down to an easily mappable and communicable concept. Both points also reemphasise the reproducibility that a composite index offers, particularly when compared to alternatives. Given their ease of creation and interpretation, and their potential to be less stigmatising than geodemographic classifications, composite indices were selected as the data reduction technique in this study.

The following section outlines the steps by which the HEAD index was developed. This follows a standard composite index formulation process, as outlined by several authors, including Lucy and Burns (2017). The subsequent sections overview the following phases: rationale for study area on which the index will be evidenced, variable selection and acquisition (including data sources), data processing, weight allocation, mapping and interpretation of results and validation.

### The Geography of the Pilot Study

Cornwall was selected as the pilot study area in this research. Being largely rural and with impressive coastline, strong surf and arts scenes, and a reputation for food and drink, Cornwall is a major UK tourist draw (Visit Cornwall, 2020). Visitors often see the county as affluent, however, it is one of the most deprived in England (Williams, 2003) receiving consistently high levels of EU social and infrastructure funding over the last 20 years (Cornwall Council, 2015; Morris, 2016). Cornwall contains 17 neighbourhoods within the 10% most deprived in the Indices of Multiple Deprivation 2019, scoring low in the Income, Employment, Education Skills and Training, Health Deprivation and Disability and Living Environment domains (Cornwall Council, 2019). Student numbers within Cornwall have been traditionally low, but have grown rapidly from 3250 in 2001/02 to 6,750 in 2008/09 (Cornwall Council, 2011, p.7), primarily due to the establishment of a joint university campus in Penryn (Davies, 2004), with HE seen as both a way to retain young people in the county and boost the local economy.

Rees et al., (2007, p.268) note that rural poverty differs from urban deprivation, particularly surrounding access to welfare and educational services, and further add that rural areas fair less favourably in spatial deprivation analyses due to lower population densities. Therefore, Cornwall’s deprived rural status, its relatively self-contained nature, and the recent growth in its HE provision makes it an interesting and ideal test bed for creating a new spatial measure for addressing higher education access. It should be noted, however, that the Isles of Scilly were omitted in this study, largely to prevent very low resident populations significantly skewing the results, and although part of the ceremonial county of Cornwall, they are independent of the mainland county (Council of the Isles of Scilly, 2020).

Putting aside the statistical arguments surrounding the robustness of POLAR, the need for a finer scale spatial targeting tool has been largely unmet since its introduction in 2005. To offer an alternative to the status quo, the Lower Super-Output Area (LSOA) census geography rather than POLAR’s MSOAs was used in this research. LSOAs typically cover 400 to 1,200 households rather than the larger MSOAs (ONS, 2011) with Cornwall having 326 LSOAs against 73 MSOAs (UK Data Service, 2020a).

## Methodology

This study follows a standard set of methodological steps observed in other similar spatial composite index studies and detailed more fully in the OECD’s Handbook on constructing composite indicators (2008). Insights gained from reviewing previous cases studies is used to inform variable selection, with acquired data then normalised and aggregated using standard index construction techniques. The index is then made ready for analysis by importing it into mapping software.

### Variable Selection and Data Acquisition

A long list of potential variables for inclusion in the index was compiled, drawing upon the aforementioned HE geodemographic projects, analyses of POLAR data and composite indicator deprivation studies. This was further supplemented by undertaking a review of studies relating to factors affecting an individual’s progression to university. The first official analysis of the Young Participation Rate (HEFCE, 2005b) was used as a starting point as this tested the measure against numerous geodemographic variables including gender, age, school performance and type, ethnicity, social class, various deprivation scores and commercial geodemographic classifications. Later reports focussed on a narrower range of variables (HEFCE, 2010a, 2014), in particular GCSE achievement, gender, parental education and occupations, income, and school type. Similarly, UCAS’ MEM tested region, birth month, a measure of rural versus urban environment and the distance from university to home in the measure’s development before concentrating on the more statistically significant gender, ethnicity, POLAR quintile, school classification, Free School Meal (FSM) indicator and IMD rank (2018). The same core variables of gender, ethnicity, and social class are seen throughout the examined studies often in combination with data from other sources. For example, in the geodemographic classification built by Singleton and Longley (2009), they feature alongside the YPR, distance from home to university, A-level scores, university course choice and school, while Kumwenda et al. (2019) combines them with parental education statistics, POLAR quintile, and FSM status to examine disadvantage’s role in choice of career specialism in medical students.

The parental education level is frequently examined, whether in relation to more educated parents seeking to gain educational advantage (Chesters, 2015), particularly through choosing schools outside their immediate locale (Allen, 2010; Rees et al., 2007; Warrington, 2005), or to determine its effect on educational attainment of their children (Cigan, 2014; Marcenaro-Gutierrez et al., 2007; Nguyen, 2016). A related variable often explored is the type of school attended with Manley and Johnston (2014) demonstrating that schools such as grammar schools that select pupils bases on academic ability perform better than both comprehensive and secondary modern schools who in turn outperform 6th form colleges. They also note that social class plays a part in this as schools situated in working class areas are less likely to send students to university. The extent of influence social class has on progression is often not clear though. Batey et al. (1999) further shows that HE progression is lower amongst areas with low income, high unemployment, and high manual occupations while those with higher income and professional occupations have an increased YPR. Harrison and Hatt (2010) also tested the inter-relationships between social class, deprivation and HE progression and found that whilst these variables are related to each other, the effect was not strong enough to be of predictive value.

The optimal list of variables was then refined as data sources were sought, with some being excluded through being unavailable through open-source channels (e.g. commercial geodemographic classifications, or university/school records) or by not being available at LSOA level (e.g. school type, Special Educational Needs, Free School Meals or GCSE/A-level attainment). The ‘Staying on in education post 16 indicator’ from the IMD’s Education Domain was identified as a proxy for GCSE Attainment based on the assumption that pupils continuing at school must have achieved some qualifications. Data for the remaining variables were downloaded from the English Indices of Deprivation (2019a), the LSOA Population Estimates Mid-2018 (2019b), or the Infuse website’s 2011 Census data (UK Data Service, 2020b), with Table 2 identifying the source used for each variable.

**Table 2:** Variable Selection and Data Sources

Variable:	Numerator:	Denominator:	Source:
Car Access	No cars or vans in household	Total households	Car or Van Availability, Census 2011, Infuse
Crime Rate	Crime Decile	N/A	Domains of Deprivation. IMD2019, ONS
Disability	Persons with Daily activities limited a little by Long-term health problem or disability + Persons with Daily activities limited a lot by Long-term health problem or disability	Total Persons	Long-term limiting illness, Census 2011, Infuse
Ethnic Minority	White and Black Caribbean + White and Black African + White and Asian + Other Mixed + Indian + Pakistani + Bangladeshi + Chinese + Other Asian + Black African + Black Caribbean + Other Black + Arab + Other Ethnicity Persons	Total Persons	Ethnic Group, Census 2011, Infuse
Gender ratio	Total Male 11 to 18 Population	Total 11 to 18 Population	Mid-2018 Population Estimates, ONS
HE Experience	Persons Aged 16 and over with their Highest Qualification Level 4 and above	Total Persons Aged16 and over	Highest level of Qualification, Census 2011, Infuse
Housing Stock	Usual Persons in Terraced + Flat; maisonette or apartment + Caravan or other mobile or temporary structure	Total Usual Persons	Accommodation type, Census 2011, Infuse
Income	Income Decile	N/A	Domains of Deprivation. IMD2019, ONS
Multiple Deprivation	IMD Decile	N/A	IMD2019, ONS
Rented accommodation	Persons Social renting + Renting from council + Private renting	Total Persons	Tenure, Census 2011, Infuse
Rurality	Geographical Barriers Decile	N/A	Sub-domains of Deprivation, IMD2019, ONS
School/Educational attainment	Staying on in education post 16 indicator	N/A	Underlying indicators, IMD2019, ONS
Single Parents	No Lone-parent households with dependent children where the lone parent is aged 16 to 74	Total households	Lone-parents, Census 2011, Infuse
Social class	Total Persons Aged 16 and over in NS-SeC Groups 4–8	Total Persons Aged 16 and over	NS-SeC, Census 2011, Infuse

### Data Processing

**Fig. 1:**
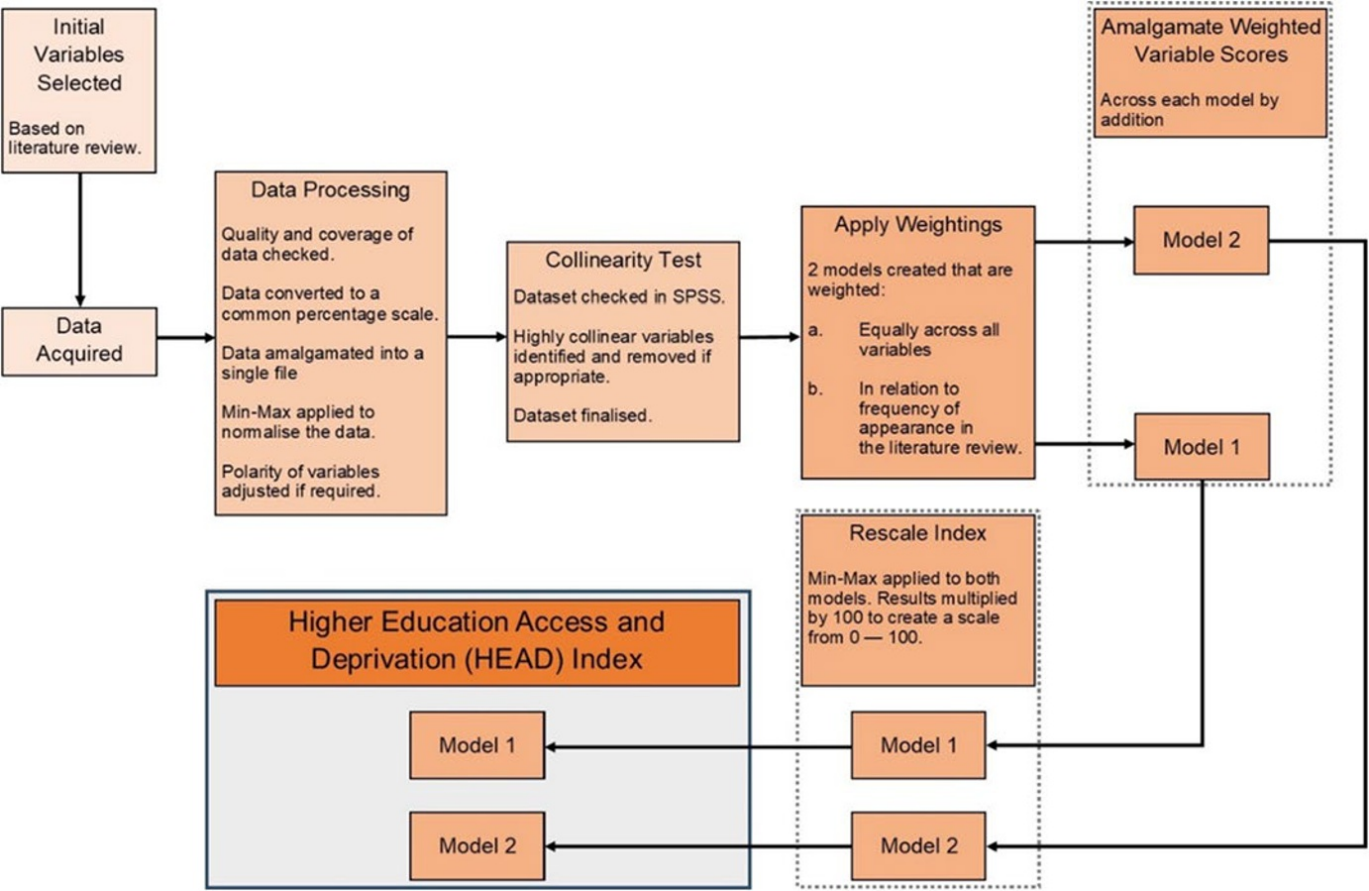
The Higher Education Access and Deprivation (HEAD) index creation process

The framework adopted in this research was developed based on two fundamental core values—simplicity and transparency. POLAR and other forms of WP data are used widely within universities, with Holland et al. (2017) identifying up to 19 different operational and strategic uses for such data, so by default any new measure needs to be understood by as wide a userbase as possible. Additionally, there is no one set method for producing a composite indicator as each stage in its creation requires decisions to be taken on different methodological approaches, e.g. the Handbook on Constructing Composite Indicators (OECD, 2008) discusses eight different mechanisms for weighting data. As the HEAD index aims to be replicable by staff who may not have in-depth spatial or statistical knowledge required for the more advanced techniques listed, this index adheres to the more simplistic techniques wherever possible. Norman et al. (2019) also emphasise the importance of simplicity when generating an index framework, particularly when seeking to encourage uptake, citing how more simple approaches tend to correlate strongly with more sophisticated measures. Transparency is also needed at every step in the composite indicator creation process (Dur & Yigitcanlar, 2015) to build confidence in using the final index as the “selecting indicators, weights, and summarising methods when constructing a composite index includes several judgement stages” (Wiréhn et al., 2015, p.70). Figure 1 gives a broad outline of the creation process used.

After downloading the datasets set out in Table 2, these were both checked visually in Excel and loaded into ArcGIS Pro to ensure complete spatial coverage with no omissions or obvious errors being discovered. Each dataset then required conversion to a common percentage scale. For census data, percentages were calculated manually in Excel with Table 2 detailing the fields used as numerators and denominators (if applicable). Similarly, the Gender ratio was manual calculated using the school age population (defined as ages 11–18 in this case) as the denominator and the number of boys in this age band as the nominator, to reflect the consistent under-representation of boys in higher education (HEFCE, 2005b, 2010a, 2017; UCAS, 2017). The ‘Staying on in education post 16 indicator’ was already expressed proportionally and required no conversion, while a lookup table was used to convert the decile values from the overall IMD score, Crime and Income Domains and Geographical Barriers sub-domains into percentage groups, similar to the methodology seen in Lucy and Burns (2017).

The data for each variable was aggregated into one file using the unique LSOA code to match the records from the different sources, before the data was normalised to render them comparable (OECD, 2008, p.15). The Min–Max method of standardisation was used to rescale the data with the lowest value data point given a value of 0 and the highest 1 using the following formulae, with ${x}_{raw}$ being the unnormalized value for conversion, ${min}_{i}$ is the lowest value found in variable ${x}_{i}$ and ${max}_{i}$ is the highest value of ${x}_{i}$.:

$$x_{norm} = \frac{{\left( {x_{raw} - min_{i} } \right)}}{{\left( {max_{i} - min_{i} } \right)\dot{ }}}$$

Finally, the dataset was checked to ensure that all variables were polarised correctly, i.e. they are measuring in the same direction and thus a high score in all variables is either favourable or not in the context of the application. Five variables required adjusting: Multiple Deprivation, Income, Crime rate, Geographical Barriers and HE Experience. The first four are based on IMD deciles where the higher the number the less deprivation exists, while HE Experience is based on persons with Level 4 qualifications rather than those without them. To adjust the directionality of these variables, each value was corrected by subtracting from 1—this then reverses the polarity of the data.

Each variable in the normalised dataset was then tested in SPSS for (multi) collinearity using the Pearson correlation coefficient to reduce the risk of any ‘double counting’ or compounding effects. This is an important step to identify any variables with particularly high correlations given a general desire for all inputs to contribute a unique dimension where possible. Of course, variables chosen to act as proxy (when combined) for a boarder concept are likely to correlate and the extent to which such correlation coefficients are used to inform final variable selection is at the discretion of the researcher. As the Income domain also contributed to the Overall IMD score and had a high collinearity (0.836), the Overall IMD score was removed from the final variable selection to reduce the risk of skewing the resulting index.

During the weighting stage, two different models were produced to determine the effect of weighting on the final indicator. Model 1 assumes all variables have the same importance and all variables were weighted equally, while in Model 2 each variable was weighted proportionally due to the frequency they occurred in the reviewed literature (Table 3). Model 2 was carried out to introduce an element of expert opinion into the model whilst also ensuring that the process is easy to follow for anybody seeking to replicate the index.

**Table 3:** Weightings used in Model 2

No. of Studies	Weighting	Variables
1	0.012	Crime Rate, Disability
2	0.024	Car Access, Housing Stock, Single Parents
3	0.036	Rented Accommodation
4	0.048	Rurality
5	0.060	Income
11	0.133	Ethnic Minority, HE Experience, School Attainment
15	0.181	Gender Ratio, Social Class

In both models, the aggregation of the variables was achieved through simply summing these together to create a total score for each LSOA. This is normally the final stage in composite indicator creation, however, to aid interpretation one last step was taken. For each model, the aggregated composite indicator scores were rescaled using the same Min–Max method employed during normalisation, with the normalised score then multiplied by 100. This puts each LSOA onto a sliding scale of 0 to 100, with 0 being the least deprived area and 100 being the most deprived with regards to education participation, making it easy for users to weigh up the relative deprivation of one area next to another, rather than interpreting raw composite indicator scores.

### Mapping the Index

**Fig. 2:**
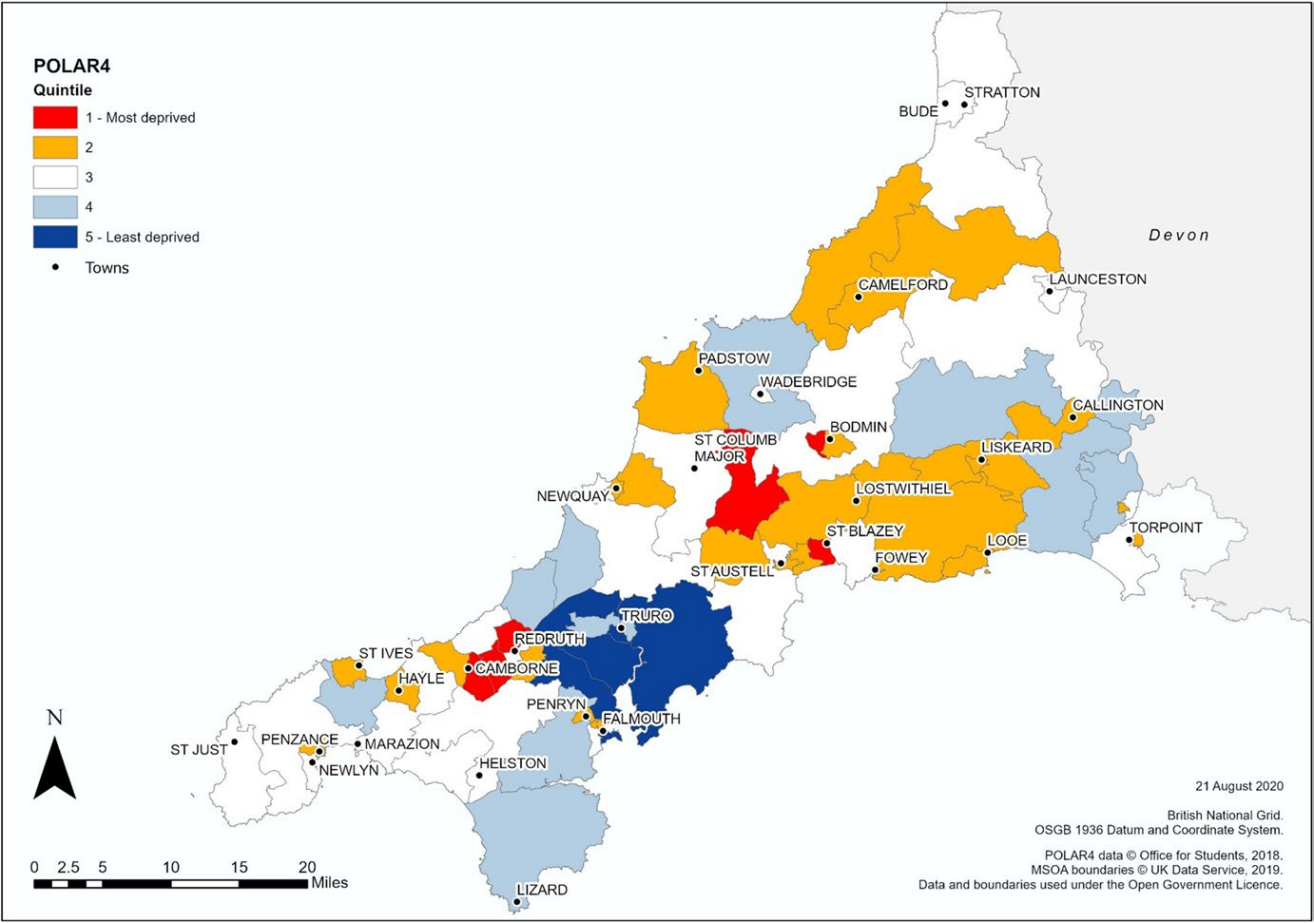
POLAR4 Higher education deprivation rates in Cornwall, by MSOA

**Fig. 3:**
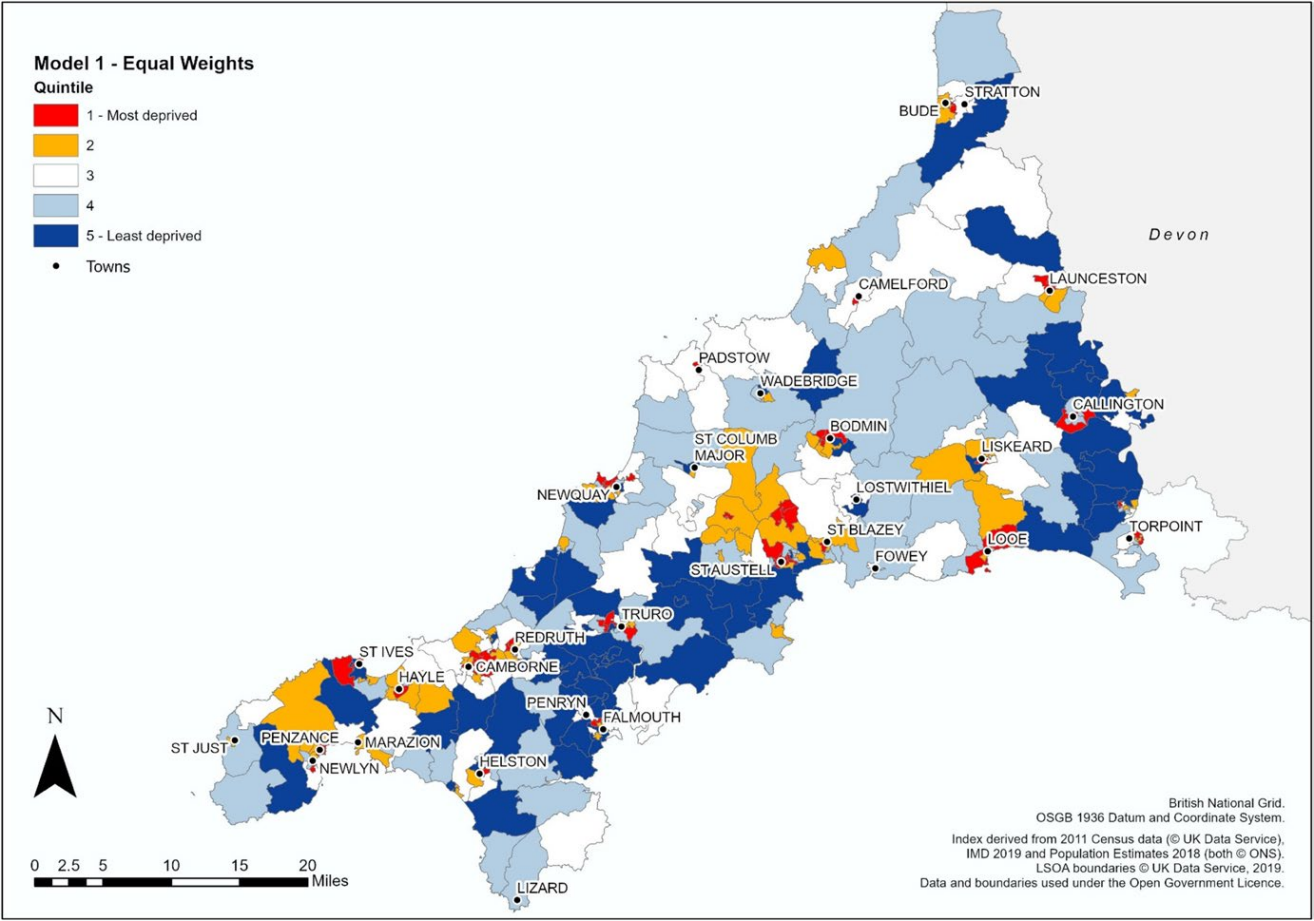
HEAD index map of Cornwall, Model 1 (equal weights), by LSOA

**Fig. 4:**
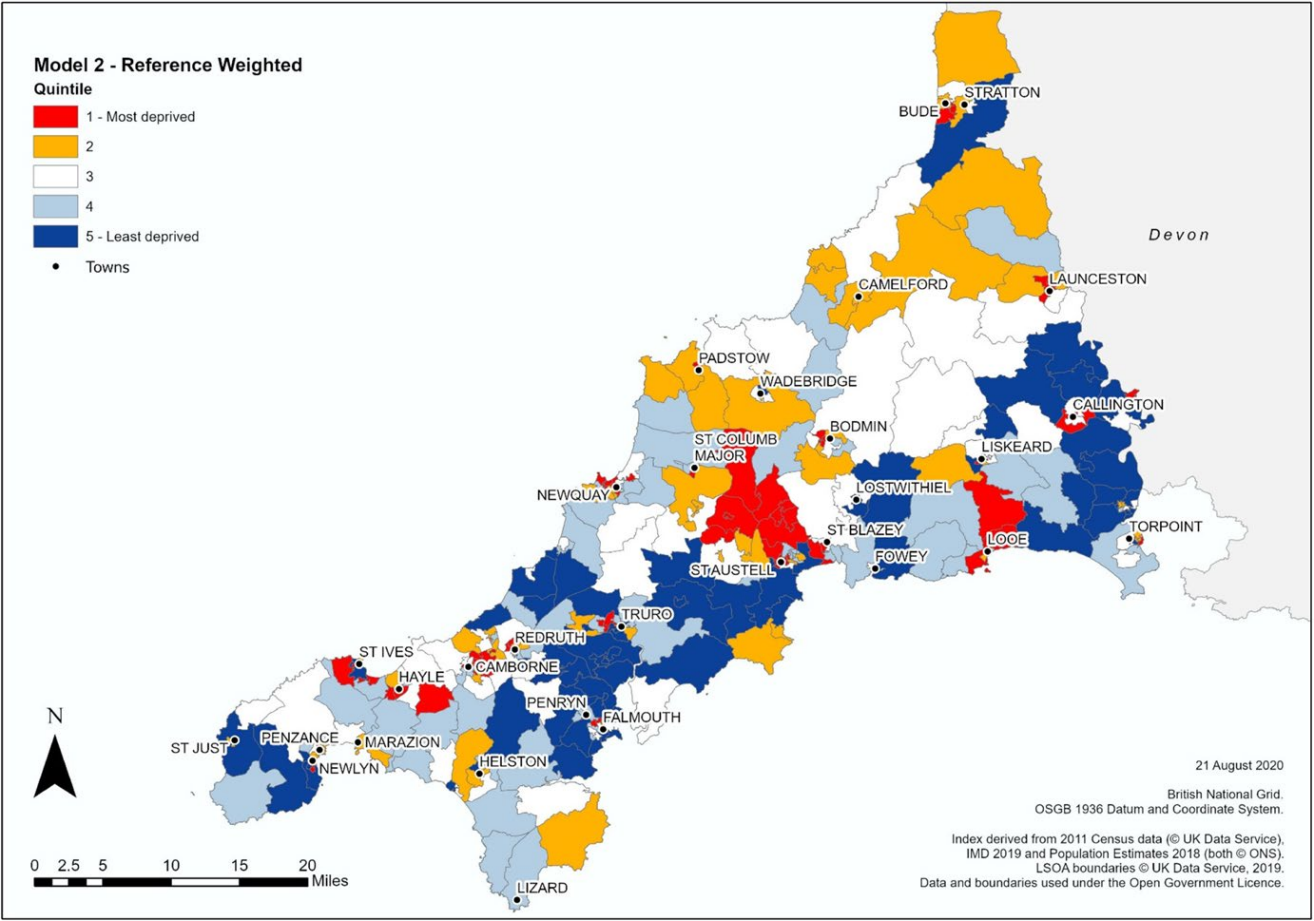
HEAD index map of Cornwall, Model 2 (weighted), by LSOA

Data for both HEAD index models were imported into ArcGIS Pro for mapping across Cornwall at LSOA level, while POLAR4 data was also loaded for mapping by MSOA. For ease of comparison, the LSOAs in both models were ranked from lowest to highest based on their overall HEAD index score and then classified using the same quintile scheme seen in POLAR data. A diverging five-class classification scheme was applied in all three datasets to display areas of low and high HE participation, with the results shown in Figs. 2, 3, 4. Under POLAR4 (Fig. 2) the areas with the lowest participation rates (quintile 1) were concentrated in Bodmin, St Blazey, Camborne, Redruth, and between St Columb Major and St Austell, while a notable north–south band of advantaged areas (quintiles 4 and 5) bisects the county from the north coast down through Truro and onwards to the Lizard. The extremes of advantage and disadvantage are firmly concentrated in the middle of the county where the largest proportion of the population resides, while areas such as north and south west of Cornwall are largely depicted as having average HE participation rates (quintile 3).

By switching resolution from MSOA to LSOA, the broad areas of homogenous HE participation rates are now replaced with a more fragmented patchwork pattern (Figs. 3 and 4 present Models 1 and 2 of the HEAD index respectively). Points of similarity still exist between the HEAD composite index versions and POLAR4 with the broad band of high HE rates across central Cornwall remaining largely intact although pockets of disadvantage now appear in Truro and Falmouth (quintile 1), and areas identified as having extremely low HE rates under such as Camborne and Bodmin continue to exist though are now much reduced in size. Both models now identify new pockets of extremely low participation (quintile 1) spread throughout the county, e.g. Newquay, St Ives, Torpoint, Callington, etc. with Model 2 identifying more “Most deprived” areas in rural environments compared to Model 1 and POLAR4 as seen to the north of Looe or the area between St Columb Major and St Austell. Both models also reclassify areas with average participation rates under POLAR4 into advantaged or disadvantages quintiles, as seen in the far north of Cornwall and near St Just in the south.

Differences exist between both HEAD index models, with Model 2 appearing to act as a compromise between POLAR4 and Model 1, preserving features from both methodologies best exemplified in comparing the area between Camelford, Launceston and Callington. Overall, 58.9% of all LSOAs remain unchanged between Model 1 and 2, with only 10 LSOAs experiencing a shift of two quintiles (3.1%) and no LSOAs experience a change of three or four quintiles (Table 4).

**Table 4:** Comparison of no. of LSOAs in Model 1 and 2 by Quintile

		Model 2					Total
	Quintile	*1*	*2*	*3*	*4*	*5*	
Model 1	1	50	15				65
	2	15	27	21	2		65
	3		19	30	12	4	65
	4		4	14	35	12	65
	5				16	50	66
	Total	65	65	65	65	66	326

The greatest consistency is observed at both ends of classification with over 75% of LSOAs in quintiles 1 and 5 remaining unchanged between Model 1 and 2, while those in the middle quintiles are more likely to shift depending on the weighting strategy employed.

**Fig. 5:**
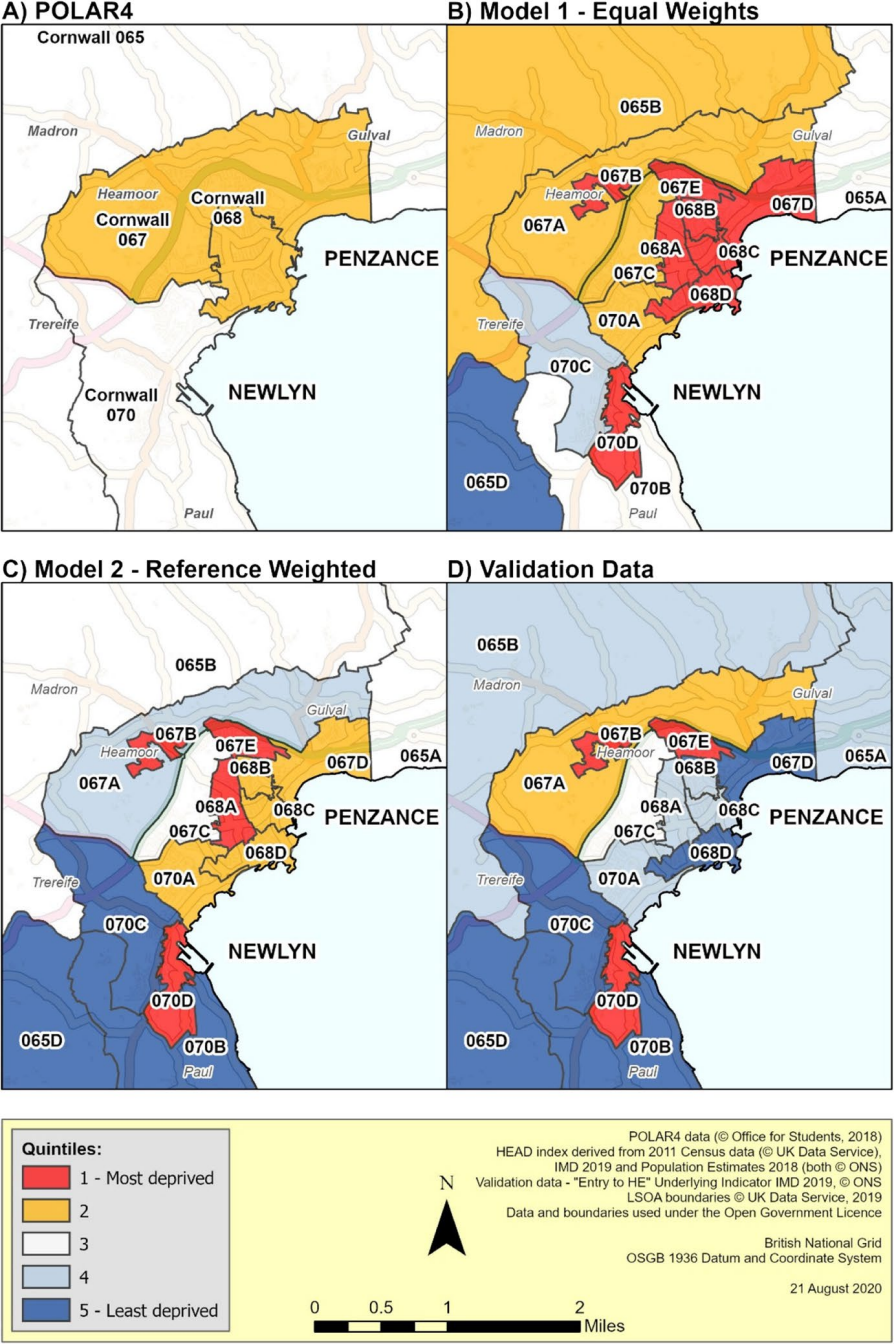
Penzance and Newlyn Detail

Figure 5 illustrates how both models and POLAR4 compare on a local level. Penzance is the retail and service hub for the Penwith Peninsula in the south west of the county while adjoining Newlyn is the centre of the Cornish fishing industry (Cornwall Council, 2016). With an estimated population on 21,313 resident in the area (Cornwall Council, 2017), the LSOAs covering ‘Newlyn Harbour and Gwavas’ and ‘Penzance St Clare and Town’ are amongst the most deprived areas identified by the Indices of Deprivation (Cornwall Council, 2019; ONS, 2019a). Under POLAR4 the area is split between quintile 3 (average HE progression) and quintile 2 (below average progression), while both composite indicator models show LSOAs (e.g., 070D, 067B and 067E) with extremely low progression LSOAs (e.g. 070D, 067B and 067E) and areas of advantage (e.g. 065D and 070C) not previously identified.

### Evaluation of the Results

To judge the overall effectiveness of the HEAD index, it was validated against the existing Entry to Higher Education Underlying Indicator which forms part of the Education, Skills and Training Domain of the IMD (ONS, 2019a). This measures the proportion of young people aged under 21 not entering HE (McLennan et al., 2019, p.40) and is therefore relevant to this study and available as a LSOA-level dataset. To provide a like-for-like comparison with the HEAD models, the indicator was normalised, ranked, and converted into quintiles using the same methods previously described. The granularity of the Entry to Higher Education Underlying Indicator resulted in this dataset being favoured over alternatives for validation. Such alternatives included: A Classification of Residential Neighbourhoods [ACORN] (CACI, 2021) and Income Deprivation Affecting Children Index [IDACI] (FFT Education Lab, 2019)—both of which are used by HEI’s as area-based proxies for deprivation and to understand (un)likely recruitment areas.

Both models and the original POLAR4 data were correlated against the validation data (Table 5), with ArcGIS Pro being used to convert the POLAR4 values from the larger MSOAs into smaller LSOAs using the in-built clip function. As this assumes all LSOAs will have the same value as the parent MSOA, this method would be problematic for in-depth spatial analysis, but was deemed acceptable here as it is intended as a rough measure of judging the fit of Models 1 and 2 against the validation data.

**Table 5:** Correlation results of HE participation indicators

Measure	Model 1	Model 2	POLAR4	Entry to HE
Model 1	1.000	0.875	0.488	0.520
Model 2	0.875	1.000	0.503	0.540
POLAR4	0.488	0.503	1.000	0.513
Entry to HE	0.520	0.540	0.513	1.000

Pearson correlation coefficient used. All correlations are significant at the 0.01 level

Both HEAD models display moderate correlation when compared to the validation data (0.520 and 0.540 respectively), and only a marginal improvement on the POLAR4 benchmark data (0.513). Differences are bound to exist between the validation data and the constructed models as the age selection criteria are not consistent across all datasets and both are measuring two separate but related concepts – one a direct count of young people going to HE from an LSOA, and the second a combined measure of the factors likely to indicate low HE progression.

The Penzance example (Fig. 5) illustrates where both the composite indicator models converge and diverge from the validation data. The aforementioned pockets of extremely low HE progression not identified by POLAR are largely consistent with the validation data as is much of the advantaged countryside surrounding Newlyn. However much of central Penzance and the north of the map diverges significantly from the validation data. As seen in comparison with POLAR4 data, Model 2 again appears to preserve some of the features of the validation data. Furthermore, as it operates well in both urban/rural environments and has a better correlation score than Model 1 it was chosen as the basis for the new HEAD index.

The results of testing against validation data highlights that this study has its limitations, which can be hopefully reduced in subsequent versions. As the published research on educational progression, widening participation, geodemographics and composite indicators continues to evolve, re-surveying the relevant literature during the lifecycle of the HEAD index should be undertaken regularly to test that decisions based on the literature, such as weighting and variable selection, are still relevant. It is important to recognise that a wider search may yield more relevant papers omitted from this review due to imprecise search criteria or unconscious research bias. Working in collaboration with other practitioners to identify the most important literature as the index is revised would further assist in reducing this risk.

While the developed framework chose a higher resolution (fine scale) areal unit to lessen problems of scale and the MAUP, these problems do not entirely disappear as LSOAs are still artificial, and even within these variations in HE progression will exist. Although not explored here, it may be possible to create a similar composite index at Output Area level and its viability is worth exploring.

The choice of variables was restricted to those that were freely available at LSOA level, adopting a difference strategy may have produced different results. Additionally, the variable selection leans heavily on the 2011 census data, which may not reflect current 2021 demographic patterns. The index will require refreshing after the 2021 UK census data is released and will require re-working in the longer term as the ONS reconfigures future national surveys (2017). There is no guarantee all of the current census fields will still be available after this change, however, switching to alternative data sources should lead to a more frequently updatable index. Likewise, not being tied to a 10-year survey cycle, with also the possibility of new variables being included that were previously unavailable, should further enrich any future iterations of the HEAD index.

The indicator construction methodology was intended to be simple, understandable, and replicable by as wide a body of potential users as possible. While the use of less complex construction steps increases the potential user base, more complex alternatives will produce different, possibly more accurate, results. Reworking the same dataset using a variety of composite indicator methodologies as detailed by the (OECD, 2008) and testing their outputs in parallel against the same validation data would be required to understand the influence of indicator construction on the results obtained.

Multiple sources of data should also be sought for validation as reliance on one source provides little frame of reference to gauge the accuracy of either the models or the validation data; a possible option includes testing against an institution’s own application records.

### Policy Implications of the HEAD Index

Since POLAR’s inception, it has become embedded within higher education and has been used in a variety of contexts including the targeting of under-represented areas, internal and external reporting, and in the distribution of financial aid to students. As the preferred measure of reporting on WP progress from the regulator, it will be challenging to persuade the sector to consider moving to a new unproven index. As such it is worth exploring the benefits and implications of adopting a composite indicator approach to spatially targeting under-representation in higher education.

The HEAD index can be used as an immediate like-for-like replacement to POLAR data in most contexts. The use of LSOAs allows for a finer targeting of outreach and support activities than previously possible, leading to staff spending time, resources, and money more efficiently. Furthermore, performance indicators and other derived measures used for internal and external reporting will have a greater degree of precision although users should remain aware of certain challenges linked to scale, as previously addressed.

In this study the HEAD index was re-categorised into quintiles, primarily to ease comparison with the existing published POLAR data. This categorisation has advantages within the current HE context as the use of POLAR quintiles is widely understood by staff so can help achieve a smooth transition to using a new measure of deprivation. However, this does not necessarily mean that quintiles are the best format for communicating or exploring spatial variations in HE progression rates. As users now have access to the underlying composite index values, they can rework and represent it for their own reporting needs work requirements, with mock-ups, surveys and focus groups being used to determine the best categorisation and symbolisation to suit individual use-cases.

The HEAD index can be incorporated into existing institutional systems and workflow processes by linking student/applicant data through a LSOA-postcode lookup table (ONS, 2016) to provide additional functionality including:

- Sending details automatically on additional support and funding available to those from the most deprived areas by collecting an enquirer’s home postcodes when they request a prospectus via a university’s website.
- The inclusion of the HEAD index as a data layer within an institutional management information system allows senior managers to monitor recruitment of students from LPNs at a university, faculty, and course level.
- Finance Departments can use the HEAD index to model the financial support required for disadvantaged students and allocate the appropriate budget to support services and faculties accordingly.

Perhaps the biggest difference between the HEAD index and the existing POLAR methodology is that through using multiple variables as an input, it allows users to directly understand the nature of the LSOAs that they are targeting through being able to examine the contributing variables. Staff can then identify areas with particular concerns and design interventions based on the underlying nature of the area(s). Potential examples include:

- LSOAs with over half their variables in quintile 1 or 2 could automatically be included in any targeted outreach programme or student support by default.
- Making bursaries or scholarships available to students from areas with low-income levels.
- Subsidising public travel to students from areas with low car ownership.
- Priority in allocating halls of residence places to students from poor housing stock areas or very rural environments.

Penultimately, it should be noted that the university sector is not homogeneous, some institutions have existed for over 500 years whereas others only gained university status in the last 10 years. Universities exist in urban, rural, and coastal environments and teach different subjects, while their Access and Participation Plans have differing emphasis on how and who they target. The weightings used in this pilot were based on the literature review results to introduce an element of expert knowledge into the index creation process and as an example of what is possible. However, a university can tailor the weightings used in indicator construction stage to match their own strategic goals or specific local environment. Furthermore, whist the framework developed here has been shown to work at a county level, it is fully scalable to encompass the whole of the UK. By undertaking additional analysis into the origins of their application data, it would also be possible for a university to create bespoke versions of the HEAD index at an institutional or faculty level based on existing recruitment patterns.

Finally, it is worth re-emphasising that the HEAD index presented in this research is the first iteration of a model designed to aid institutions with their approach to recruitment. Following the publication of the England and Wales 2021 Census data, the model will be recalculated, and the results contrasted with those from 2011. A similar approach will be taken for Scotland once data are available post-2022.

## Conclusion

Whilst the geographical targeting of under-represented students by universities is both important and achievable, several assumptions and limitations remain regardless of the methodology or datasets chosen. Issues surrounding scale, the use of appropriate boundaries and the ecological fallacy continue to be present in the HEAD index although these have been partially addressed through moving to a finer level scale (areal unit) and adopting a multivariate approach. Recently the OfS launched their new experimental spatial measure ‘Tracking Underrepresentation by Area’ (TUNDRA), at LSOA level (OfS, 2020a), which measures the rate state-school students progress onwards with their education. Whether this new trial measure will replace POLAR remains to be seen. Whilst the move to a finer resolution addresses some of the criticisms aimed at its predecessor, by neglecting the underlying properties of the areas it does not fully embrace the benefits gained through being able to interrogate the underlying data gained through using a composite index approach.

Higher education in the UK is currently operating in a complex unstable environment. Brexit has seen a drop in applications to courses from EU countries (Weale, 2019), whilst the COVID-19 global pandemic is affecting applications from international students in a similar way (Savage, 2020) and both are likely to cause some university’s financial difficulties (Burns, 2020). While UCAS reported that during the first UK lockdown a record number of ‘home-rated’ (UK) students had applied to degree courses (UCAS, 2020), school pupils were unable to sit exams due to the pandemic and a grading algorithm was used to predict their A-level results. With the release of the results a large proportion of students found their expected results considerably downgraded affecting their chances of entering university (Adams, 2020). Students from disadvantaged areas were disproportionally affected by the algorithm (Adams & McIntyre, 2020) with the general public seeing the HE sector’s response to this crisis as inflexible and elitist (Weale, 2020). This stance was reflected in institutions’ responses earlier in summer (2020) during the in-campus UK Black Lives Matter demonstrations (Batty, 2020) with protesters demanding the decolonisation of teaching and the removal of problematic historical reminders from universities (Mohdin, 2020). At present, it is not clear what long-term effect this uncertainty will have on the UK higher education sector, however, if universities wish to both increase their recruitment and avoid negative press there are worse strategies than fully engaging in widening participation by recruiting students from under-represented areas and designing appropriate support to enable them to succeed in their studies.
